# Effects of AgRP Inhibition on Energy Balance and Metabolism in Rodent Models

**DOI:** 10.1371/journal.pone.0065317

**Published:** 2013-06-06

**Authors:** Roxanne Dutia, Andrea J. Kim, Matthew Modes, Robert Rothlein, Jane M. Shen, Ye Edward Tian, Jumana Ihbais, Sam F. Victory, Carmen Valcarce, Sharon L. Wardlaw

**Affiliations:** 1 Department of Medicine, Columbia University College of Physicians & Surgeons, New York, New York, United States of America; 2 TransTech Pharma, Inc., High Point, North Carolina, United States of America; University of Texas Health Science Center at San Antonio, United States of America

## Abstract

Activation of brain melanocortin-4 receptors (MC4-R) by α-melanocyte-stimulating hormone (MSH) or inhibition by agouti-related protein (AgRP) regulates food intake and energy expenditure and can modulate neuroendocrine responses to changes in energy balance. To examine the effects of AgRP inhibition on energy balance, a small molecule, non-peptide compound, TTP2515, developed by TransTech Pharma, Inc., was studied *in vitro* and in rodent models *in vivo*. TTP2515 prevented AgRP from antagonizing α-MSH-induced increases in cAMP in HEK 293 cells overexpressing the human MC4-R. When administered to rats by oral gavage TTP2515 blocked icv AgRP-induced increases in food intake, weight gain and adiposity and suppression of T4 levels. In both diet-induced obese (DIO) and leptin-deficient mice, TTP2515 decreased food intake, weight gain, adiposity and respiratory quotient. TTP2515 potently suppressed food intake and weight gain in lean mice immediately after initiation of a high fat diet (HFD) but had no effect on these parameters in lean chow-fed mice. However, when tested in AgRP KO mice, TTP2515 also suppressed food intake and weight gain during HFD feeding. In several studies TTP2515 increased T4 but not T3 levels, however this was also observed in AgRP KO mice. TTP2515 also attenuated refeeding and weight gain after fasting, an effect not evident in AgRP KO mice when administered at moderate doses. This study shows that TTP2515 exerts many effects consistent with AgRP inhibition however experiments in AgRP KO mice indicate some off-target effects of this drug. TTP2515 was particularly effective during fasting and in mice with leptin deficiency, conditions in which AgRP is elevated, as well as during acute and chronic HFD feeding. Thus the usefulness of this drug in treating obesity deserves further exploration, to define the AgRP dependent and independent mechanisms by which TTP2515 exerts its effects on energy balance.

## Introduction

The hypothalamic melanocortin system is a critical regulator of energy homeostasis in both animals and humans. This system is comprised of proopiomelanocortin (POMC) and agouti-related peptide (AgRP) neurons, located in the arcuate nucleus of the hypothalamus, which project to second-order neurons that express melanocortin receptors (MC-R); the peptide products of these neurons interact at the MC-R to regulate energy balance [1], [2]. POMC-derived α-melanocyte-stimulating hormone (MSH) is an agonist for the G-protein coupled MC4-R that reduces food intake and increases energy expenditure (EE) [3]–[6]. Oppositely, AgRP behaves as a potent melanocortin receptor antagonist, and in some reports, an inverse agonist, that promotes positive energy balance [7]–[10]. Both neurons are targets for leptin and insulin and can integrate a variety of nutritional and neuronal signals to regulate energy balance [1].

The role of AgRP in the regulation of energy balance has been examined in a number of different rodent models. AgRP expression in the arcuate nucleus is increased during fasting and in mice with genetic leptin deficiency [11]–[13]. Both overexpression of AgRP and intracerebroventricular (icv) AgRP administration increase food intake, body weight and adiposity, and AgRP also decreases EE [7], [8], [14], [15], and can mediate some of the neuroendocrine responses to food deprivation, including suppression of the hypothalamic-pituitary-thyroid (HPT) axis [8], [16]–[18].

In addition, activation of AgRP neurons using designer receptors rapidly induces voracious feeding, decreases EE and increases adiposity [19]. While reduction of hypothalamic AgRP expression by RNA interference has been shown to increase metabolic rate and decrease body weight [20], genetic deletion of AgRP yields much milder effects, implicating developmental compensation [18], [21]. Postembryonic ablation of AgRP neurons in mice leads to starvation in adults but not neonates [22], however the starvation phenotype appears to be due to the loss of γ-aminobutyric acid (GABA) from AgRP neurons, rather than the loss of AgRP peptide [23]. To date, there have been no reports of a specific pharmacological inhibitor of the AgRP peptide. Such an inhibitor could be used to help elucidate the extent to which AgRP contributes to energy homeostasis and neuroendocrine responses to food deprivation. An AgRP inhibitor also has the potential to be utilized in a therapeutic capacity, particularly under conditions in which AgRP is elevated. The drug development of MSH agonists has been complicated by a number of side effects, including hypertension, possibly related to the extensive projection of POMC neurons and wide distribution of brain MC-Rs [24]–[28]. In contrast, AgRP neuronal projections are more limited to brain areas involved with energy balance, making AgRP an attractive target for pharmacological inhibition [27]–[29].

TransTech Pharma, Inc. has developed a series of potent, small molecular weight, non-peptide, compounds that selectively inhibit AgRP’s antagonist activity. These compounds displayed high brain penetration after oral administration, and when studied *in vitro* prevented AgRP from antagonizing MC4-R signaling. Studies on one of these compounds showed that it was specific for AgRP, as it did not block the ability of SHU9119, a synthetic MC3/4-R antagonist, to antagonize MC4-R signaling. Furthermore, there was no direct interaction of this compound with the MC4-R, either alone or in the presence of α-MSH. One of these compounds, TTP2515, was used for the current studies. We utilized TTP2515 in numerous rodent models to further investigate the contribution of AgRP to energy balance. We initially examined the ability of TTP2515 to inhibit the effects of exogenous icv AgRP administration in the rat. We next investigated the ability of this compound to inhibit endogenous AgRP in lean and obese mouse models, including diet-induced obese (DIO) and leptin-deficient *ob/ob* mice. As the melanocortin system is known to mediate adaptive responses observed after acutely switching mice to a high fat diet (HFD), we investigated the effects of TTP2515 under these conditions [30]. As AgRP is markedly upregulated by fasting, we also studied the metabolic responses to fasting and refeeding [11], [13], [31]. Finally, we examined the effects of TTP2515 on the thyroid axis under both fed and fasted conditions in a number of these experiments. AgRP KO mice were employed in these various models to determine which effects of TTP2515 were specifically due to AgRP antagonism.

## Methods

### Ethics Statement

All animal experiments were performed at Columbia University in approved animal facilities in compliance with federal regulations governing the use of animals in research. The animal facilities at Columbia University are accredited by the Association for the Assessment and Accreditation of Laboratory Animal Care and staffed by full time veterinarians (Animal Welfare Number A3007-01). All studies were in compliance with The National Research Council’s Guide to the Care and Use of Laboratory Animals and were approved by the Columbia University Institutional Animal Care and Use Committee.

### Experiments

A streamlined description of all of the experiments is provided in **Table S1.**

#### In vitro

**Figure 1:**
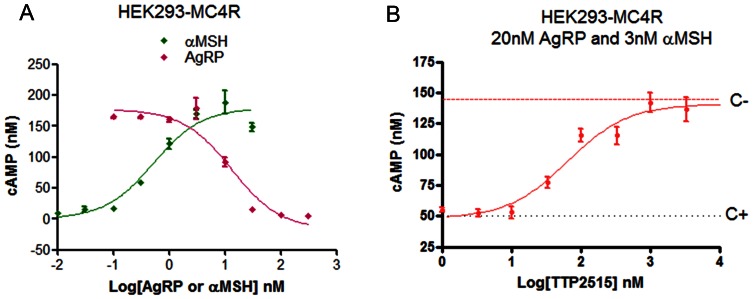
Effects of AgRP and TTP2515 on α-MSH-induced cAMP production. (**A)** α-MSH-induced cAMP production in HEK293 cells overexpressing human MC4R (green diamond); inhibition of α-MSH(3 nM)-induced cAMP production by increasing amounts of AgRP (pink diamond) (**B)** α-MSH-induced cAMP production in HEK293 cells overexpressing human MC4-R in the presence of 20 nM AgRP, 3 nM α-MSH and increasing concentrations of TTP2515 (red diamond). (C+, cAMP production in the presence of α-MSH (3 nM) and AgRP (20 nM) and in the absence of TTP2515. C-, cAMP production in the presence of α-MSH (3 nM) no AgRP or TTP2515).

The effect of TTP2515 on AgRP inhibition of the MC4-R was tested *in vitro* in HEK293 cells overexpressing the human MC4-R. The cell line was licensed from the University of Michigan [32]. The cells were cultured in Gibco Dulbecco’s Modified Eagle Medium (Life Technologies; Grand Island, NY USA) medium supplemented with 0.1 mM non essential amino acids and 10% fetal bovine serum and kept at 37°C in a humidified atmosphere containing 5% CO_2_ until they were 65–80% confluent. On the day of the experiment the cells were harvested and resuspended to a density of 2×10^6^ cells/ml in Earle’s Balanced Salt Solution (Sigma-Aldrich; St. Louis, MO, USA, containing 1∶10 dilution of anti-cAMP antibody). 20×10^3^cells/well were added to 384-well Nunc plates containing human AgRP _83–132_ (20 nM final concentration) and TTP2515 (at the final assay concentrations depicted in Fig. 1). After 15 minutes of incubation at 37°C in a humidified atmosphere containing 5% CO_2_, the cells were stimulated with α-MSH (12 nM final assay concentration) and kept under the same incubation conditions for 30 minutes. At the end of the incubation period, cAMP production was measured using a [FP]2 cAMP fire kit (Perkin-Elmer Cat # FB A203040KT) according to the manufacturer instructions. Positive (C+, 20 nM human AgRP_83–132_ without compound) and negative (C-, no AgRP and no compound) controls were run in parallel under the same experimental conditions.

#### Rats

Male Sprague-Dawley rats weighing 200–250 g were purchased from Charles River (Wilmington, MA, USA) and used for all rat experiments. Animals had *ad libitum* access to water and LabDiet® Rodent Chow 5001 (13.5% fat; PMI Nutrition International). Rats were acclimatized to a natural light/dark cycle prior to surgery. In all experiments, rats were anesthetized with pentobarbital (50 mg/kg) by intraperitoneal (i.p.) injection for icv cannula placement. In the acute icv injection rat experiment, a 22-gauge stainless steel cannula was inserted stereotaxically into the right lateral ventricle (coordinates from bregma: lateral 1.3 mm; caudal 0.8 mm; depth from dura 3.5 mm). In the chronic infusion experiment, a 28-gauge stainless steel cannula connected by vinyl catheter tubing to a 7-day osmotic pump (ALZET model 2001, Cupertino, CA, USA) delivering 1 µl/hr of normal saline was inserted stereotaxically into the right lateral ventricle (same coordinates as above). Rats were individually housed and allowed to recover for 5–7 days after cannula placement. During this recovery period, rats receiving icv injections were acclimated to restraining to minimize stress during injections. Before each experiment, rats were divided into treatment groups of equivalent weight and daily food intake. In all experiments peptides were dissolved from lyophilized stock in sterile normal saline immediately before use. Animals exhibiting signs of illness and whose food consumption fell to less than 10 grams per day were excluded from analyses. Two rats from *Experiment 2a* were excluded from day 3 onwards due to illness and decreased food intake.

#### Mice

Mice were housed under barrier conditions with a 12∶12-hr light-dark cycle. Male C57BL/6 mice from Charles River Laboratories and C57BL/6J mice and leptin-deficient mice (Stock #632) from Jackson Laboratories (Bar Harbor, ME, USA) were utilized in the experiments as indicated. The AgRP knockout (KO) mouse line was obtained from Dr. Van der Ploeg [21]. Mice were backcrossed 6 times to a coisogenic C57BL/6J line, the C57BL/6J-*A^w-J^*/J strain. AgRP KO and WT mice were generated from homozygous matings and male mice were used in the experiments as indicated. Ablation of AgRP in the mediobasal hypothalamus was confirmed by genotyping and via both qRTPCR for gene expression (*F5′GCAAAGGCCATGCTGACTGC3′, R5′CTTCTTGAGGCCATTCAGAC3′*) and via two separate assays measuring AgRP (Human AgRP Quantikine ELISA, R&D Systems, Inc, Minneapolis, MN; and RIA, using synthetic AgRP_83–132_ standard and iodinated tracer as described previously [33]).

#### Mouse body weight, food intake, and body composition

All body weight and food intake measurements were obtained daily from singly housed mice unless otherwise indicated. Food was measured using either the LabMaster Calorimetry System feeding baskets or specialized feeding chambers that fit into traditional homecages. Body composition measurements were obtained by NMR (Minispec Analyst AD; Bruker Optics, Silberstreifen, Germany). In all experiments, baseline body weight, food intake and body composition were equivalent between groups, unless otherwise indicated.

#### Mouse diets

The following diets were used for mouse experiments as indicated: normal chow diet (Lab diet #5053, 13% kcal from fat), breeder chow diet (Lab diet #5058, 22% kcal from fat), 45% HFD (Research Diets 12451, 45% kcal from fat), 60% very HFD (Research Diets 12492, 60% kcal from fat), and 10% chow diet (Research Diets 12450B, 10% kcal from fat).

#### Indirect calorimetry

The LabMaster Calorimetry System (TSE Systems, Bad Homburg, Germany) was used to measure oxygen consumption (VO2), carbon dioxide production (VCO2) and activity every 26 minutes (except for *Experiment 5,* which was every 14 minutes). Mice were acclimated to the calorimetry system for a minimum of 24 h prior to starting the experiment. After acclimation, baseline measurements were obtained for a minimum of 24 h to appropriately randomize mice. In all experiments, baseline calorimetry parameters were equivalent between groups. Approximately 2 h of measurements were excluded during each dosing session as the mouse was physically removed from the chamber for gavaging. Relative VO2 and energy expenditure (EE) were derived by dividing the mean VO2 or EE of a specific time period (ie-dark cycle) by the total counts of that time period; values are presented as multiplied by 1000.

#### Drug compound

In all animal experiments, TTP2515 was dissolved in sterile water and administered via oral gavage at the dosage indicated (5–50 mg/kg). The control group received water at the same volume per kg as the treatment group. Animals were acclimated to gavaging before experimental treatment was administered. In the all experiments except *Experiment 2a,* TTP2515 was administered twice daily, with doses 10–12 h apart.

#### Hormone analyses

Blood was collected from the trunk after decapitation at sacrifice or from the submandibular pouch. Trunk blood was collected into tubes containing EDTA and plasma was stored at −80°C. Submandibular blood samples were collected into tubes at room temperature and serum was stored at −80°C. Hormones were measured using commercially available RIAs: Leptin and insulin (EMD Millipore, Billerica, MA, USA) and total T4, free T4 and total T3 (Coat-A-Count ®, Siemens Healthcare Diagnostics, Tarrytown, NY, USA).

#### Measurement of hypothalamic mRNA levels

RNA isolation was performed using the RNeasy Lipid Tissue Mini Kit (Qiagen USA, Valencia, CA) in conjunction with the RNase-Free DNase set (Qiagen USA). cDNA was synthesized using the Superscript III First-Strand cDNA Synthesis Kit (Life Technologies Corporation/Invitrogen, Grand Island, NY) and was analyzed using quantitative RT-PCR performed with Lightcycler 480 SYBR Green I Master (Roche Applied Science, Indianapolis, IN) in the Lightcycler 480 Real-Time PCR system (Roche Applied Science). Samples were normalized to β-actin. Primer sequences: Mouse: *Pomc* F5′CAGTGCCAGGACCTCACCACGG3′ R5′ CGGTCCCAGCGGAAGTGACCC3′; *Agrp* F5′ GCAAAGGCCATGCTGACTGC3′ R5′ CTTCTTGAGGCCATTCAGAC3′. Rat: *Pomc* F5′CAGTGCCAGGACCTCACCACGG3′ R5′CGGTCCCAGCGGAAGTGACCC3′; *Agrp* F5′CATGCCCTAGCTACAGGAAG3′ R5′ GCAGTGCCAGCAGGACA3′.

### Statistical Analysis

Statistical analysis was performed with Student’s *t* test when only two groups were compared. Analysis of variance (ANOVA) followed by Fisher’s protected least squares difference test was used when comparing more than two groups. Repeated measures two-way ANOVA with Bonferroni post-hoc analysis or Dunnett’s multiple comparison test was used to analyze cumulative food intake and body weight measurements in multiple groups over time. *P*<0.05 was considered statistically significant. Results are reported as mean values ± SEM.

### Experimental Protocols

#### Experiment 2: Effects of TTP2515 on the orexigenic and metabolic effects of icv AgRP injection in rats

In *Experiment 2a*, four groups of rats were studied (n = 5–7/group). Half received icv human AgRP_83–132_ (Phoenix Peptides 003–53; Burlingame, CA, USA) with either oral TTP2515 or water and half received icv saline with either oral TTP2515 or water. On day 0, between 1730–2030 h, rats received either oral TTP2515 (30 mg/kg) or water and either 0.2 nmol AgRP or saline icv (delivered in a 5 uL bolus over the course of one minute). The following day (day 1), rats received either TTP2515 (30 mg/kg) or water between 1130–1230 h. On day 2 rats received a final dose of oral TTP2515 or water and icv AgRP or saline between 1500–1800 h. Measurements were obtained on days 1–4 between 0900–1000 h and rats were sacrificed on day 4. In *Experiment 2b*, four groups of rats were used to study the effects of TTP2515 during chronic icv AgRP infusion (n = 7–8/group). On day 0, icv pumps infusing saline were exchanged for new pumps delivering either saline or 0.64 nmol/day human AgRP_83–132._ The first oral dose of water or TTP2515 (30 mg/kg) was administered shortly after the pump exchange, and water or TTP2515 continued to be administered twice daily between 800–1000 h and 1800–2000 h. Rats were sacrificed on day 8. Trunk blood was collected for hormone assays, a 3-mm mediobasal hypothalamic section was dissected for mRNA analysis, and adipose and liver weight were quantified.

#### Experiment 3: Effects of TTP2515 on metabolic parameters in DIO mice

In *Experiment 3a,* 8-week old C57BL/6 mice received a 45% HFD for 16 weeks at which time mean body weight increased from 21.1±0.3 g to 39.4±1.0 g. Mice were randomized to receive either water or TTP2515 (30 mg/kg) by oral gavage twice daily for 21 days (n = 7–8/group). Body weight and food intake were monitored approximately every 3 days. On day 15, blood was obtained after a 6 h fast. Body composition was assessed before treatment and on day 20 of treatment. Mice received the last dose of TTP2515 or water at 0800 h on day 21 and were sacrificed 6 h later. At sacrifice, blood was collected for hormone analyses, a 3-mm mediobasal hypothalamic section was dissected for mRNA analysis, and liver weight was quantified. In *Experiment 3b,* 6-week old C57BL/6J mice received a 45% HFD for 15 weeks during which time mean body weight increased from 21.5±0.3 g to 34.6±0.5 g. Mice were acclimatized to calorimetry chambers and received treatment of either TTP2515 (30 mg/kg) or water twice daily starting during the dark cycle and continuing for 3.5 days (n = 8/group).

#### Experiment 4: Effects of TTP2515 on metabolic parameters in leptin-deficient mice

9-week old leptin-deficient *ob/ob* mice maintained on a normal chow diet were randomized to receive water or TTP2515 twice daily at increasing doses (*Days 1–4*, 5 mg/kg; *Days 5–7*, 15 mg/kg; *Days 8–10,* 30 mg/kg; *Days 11–25*, 50 mg/kg) for a total of 25 days (n = 7/group). Mice were placed in calorimetry chambers during days 1–14. Mice were fasted before the evening of day 13 and food intake was measured during refeeding the next morning. A second fast was performed before the evening of day 19 for an i.p. glucose tolerance test (GTT). Body composition was assessed before treatment and on day 24 of treatment. Mice received the last dose of TTP2515 or water on day 24 at 2200 h and were sacrificed at 1100 h on day 25. At sacrifice, blood was collected for hormone analyses and liver weight was quantified.

#### Experiment 5: Effects of TTP2515 on metabolic parameters in fed and fasted lean BL6 mice

16-week old C57BL6/J mice on a breeder chow diet were administered either TTP2515 (30 mg/kg) or water twice daily in calorimetry chambers (n = 8/group). Mice were fed on days 1–3 and then fasted. Mice received the last dose of TTP2515 or water at 2100 h on day 3 and were sacrificed at 0800 h on day 5 and blood was collected for hormone analyses.

#### Experiment 6: Effects of TTP2515 on metabolic parameters in AgRP KO and WT mice on a 45% fat diet and calorimetry parameters in AgRP KO mice

In *Experiment 6a,* 20-week old AgRP KO and WT mice maintained on breeder chow were switched to a 45% HFD (n = 6–7/group). The next day (24 h later, referred to as day 1) mice started treatment with either water or TTP2515 (30 mg/kg) in the AgRP KO groups or TTP2515 in the WT group, twice daily for 20 days. Body composition was assessed before treatment and on day 19 of treatment. Mice received the last dose of TTP2515 or water on day 20 at 0900 h and mice were sacrificed 6 h later, and blood was collected for hormone analyses. In *Experiment 6b,* 5–7 month old AgRP KO mice maintained on a breeder chow diet received either TTP2515 at increasing doses (*Days 1–7*, 30 mg and *Days 7–10*, 50 mg/kg) or water for 10 days in calorimetry chambers (n = 8/group).

#### Experiment 7: Effects of TTP2515 on hyperphagia and metabolic parameters after an acute switch to a very high fat diet in C57BL6/J mice and AgRP KO mice

In *Experiment 7a,* 14-week old C57BL6/J mice maintained on a breeder chow diet were switched to a 60% very HFD and simultaneously started treatment with either TTP2515 (30 mg/kg) or water twice daily for 5 days (n = 8/group). Body composition was assessed on day 4 of treatment. Mice received their last dose of TTP2515 or water at 0800 h on day 6 and were sacrificed 4 h later. At sacrifice, blood was collected for hormone analyses and a 3-mm mediobasal hypothalamic section was dissected for mRNA analysis. In *Experiment 7b,* 6–7 month old AgRP KO mice maintained on a 10% chow diet were switched to a 60% very HFD and simultaneously started treatment with either TTP2515 (5, 15 or 30 mg/kg) or water for 7 days (n = 7/group). Body composition was assessed at baseline and on day 4 of treatment. On the evening of day 4, mice received TTP2515 or water at 2000 h. On day 5, submandibular pouch blood was collected at 1100 h and mice continued to receive TTP2515 or water twice daily with the last dose at 2200 h on day 6. Mice were fasted starting at 2100 h on day 5 and blood was collected at 0800 h on day 7.

#### Experiment 8: Effects of TTP2515 on refeeding and T4 after fasting in AgRP KO and WT mice

In *Experiment 8a,* 5–7 month old AgRP KO and WT mice (n = 7–8/group) maintained on a normal chow diet were utilized in three separate fasting and refeeding experiments. In all experiments, both AgRP KO and WT mice were fasted for 24 h and then started treatment with either TTP2515 (10, 25, or 50 mg/kg) or water. Mice were refed 1 h after the first dose. Food intake and body weight measurements were obtained at the 3, 9, and 20 h timepoints and a second dose of TTP2515 or water was administered at the 9 h timepoint. In *Experiment 8b,* 3–4 month old AgRP KO and WT mice maintained on normal chow diet were utilized in this experiment (n = 7–9/group) to measure T4 levels after fasting. Before intervention, submandibular pouch blood was collected for baseline values. After recovery, mice were fasted and received 3 doses of TTP2515 or water at 2100 h on day 1, and at 0900 and 2000 h on day 2 and another blood sample was obtained at 0700 h on day 3.

## Results

### Experiment 1: Effects of TTP2515 *in vitro*

HEK293 cells overexpressing the human MC4-R were used to study the effects of TTP2515 on AgRP inhibition of α-MSH-induced cAMP production. As expected, α-MSH increased cAMP production and AgRP inhibited α-MSH-induced cAMP production by competing with α-MSH for binding to the MC4-R (**Fig. 1A**). TTP2515 blocked AgRP inhibition of α-MSH-induced MC4-R activation; with TTP2515 α-MSH was able to activate the MC4-R even in the presence of AgRP (**Fig. 1B**). Furthermore, TTP2515 had no effect on cAMP levels in HEK293 cells expressing MC4-R in the absence of AgRP, either with or without sub-maximal concentrations of α-MSH (results not shown).

### Experiment 2: Effects of TTP2515 on the Orexigenic and Metabolic Effects of icv AgRP Injection in Rats

#### Experiment 2a

**Figure 2:**
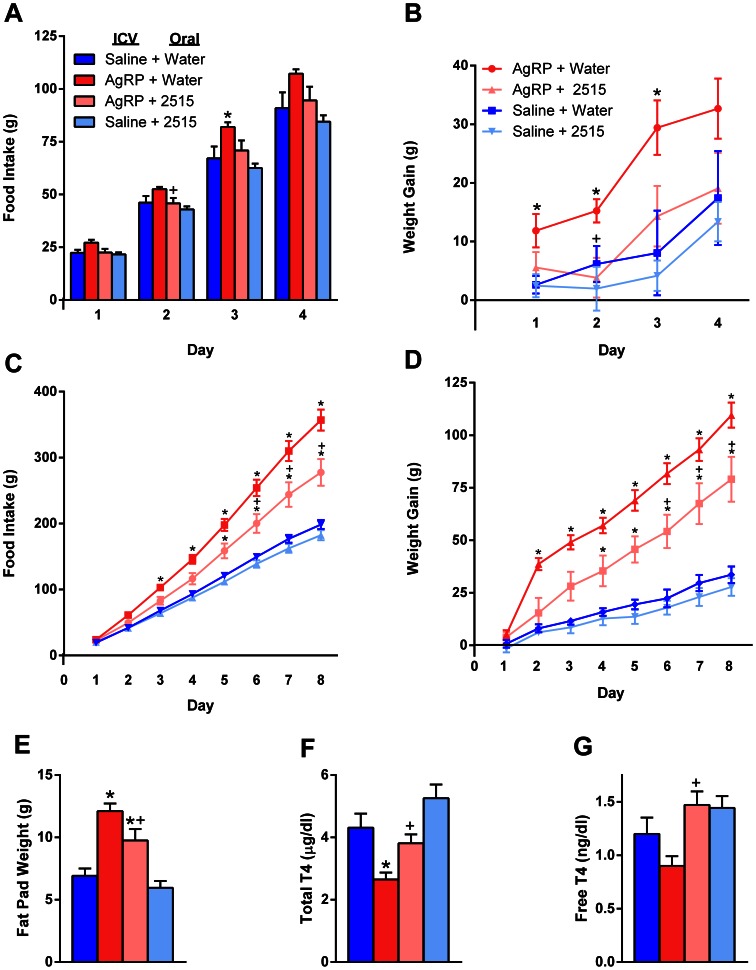
Effects of TTP2515 on body weight and food intake in rats receiving icv AgRP. (**A–B**) Rats received a single icv injection of 0.2 nmol AgRP or saline on days 0 and 2 and received 30 mg/kg TTP2515 or water via oral gavage once per day during days 0–2. (**A,B**) AgRP+Water increased food intake and body weight gain; this was partly reversed in the AgRP+TTP2515 group. (**C–G**) In a separate experiment, rats were continuously infused icv with either 0.64 nmol/day AgRP or saline and gavaged twice daily with either water or TTP2515 at 30 mg/kg. (**C,D**) AgRP significantly increased food intake and body weight gain during the entire treatment period. The AgRP-induced increases in food intake and body weight gain were significantly attenuated by TTP2515. (**E**) Fat pad mass increased with AgRP and was attenuated by TTP2515. (**F**) Total T4 levels were significantly suppressed in the AgRP+Water group and this suppression was almost entirely reversed in the AgRP+TTP2515 group. (**G**) Free T4 levels tended to be suppressed in the AgRP+Water group (p = .08 vs Saline+Water) and this suppression was reversed in the AgRP+TTP2515 group. *p<.05 vs Saline+Water; ^+^p<.05 AgRP+Water vs AgRP+TTP2515.

*R*ats received either a single icv injection of AgRP or saline on days 0 and 2, and a single oral gavage of TTP2515 (30 mg/kg) or water on days 0, 1 and 2. Overall during days 1–2, concomitant treatment with TTP2515 significantly attenuated AgRP-induced increases in food intake and body weight (p<0.05, **Fig. 2A,B** ). On day 3, food intake and body weight gain were significantly increased in the AgRP+Water group (p<0.05), and these increases tended to be attenuated in the AgRP+TTP2515 group (p = 0.06 vs AgRP+Water) so that this group was not different from saline. No significant differences between the Saline+Water and Saline+TTP2515 groups were observed during the entire experiment.

#### Experiment 2b

Rats were continuously infused icv for 8 days with either AgRP or saline and also received twice daily oral gavage with either TTP2515 (30 mg/kg) or water. AgRP significantly stimulated food intake and body weight gain during the entire experiment (p<0.05). Overall, AgRP-induced increases in food intake and body weight gain were partly reversed in the AgRP+TTP2515 group (p = 0.01**,** **Fig. 2C,D** **)**. At sacrifice, liver weight **(Table 1)** and fat pad mass (**Fig. 2E**) were significantly increased in rats receiving AgRP (p<0.0001), however these increases were attenuated in the AgRP+TTP2515 group (p<0.05 vs AgRP+Water). Total T4 levels were significantly suppressed by AgRP+Water treatment (p<0.01 vs Saline+Water, **Fig. 2F**) and this was reversed in the AgRP+TTP2515 group (p<0.05). Free T4 levels tended to be suppressed in the AgRP+Water group (p = 0.08 vs Saline+Water, **Fig. 2G**) and this was reversed in the AgRP+TTP2515 group (p<0.01). No differences in total T3 levels were detected (**Table 1**). Leptin and insulin levels were significantly elevated in both groups receiving icv AgRP, and concomitant TTP2515 treatment tended to decrease levels of these hormones, however this did not reach significance (**Table 1**). Hypothalamic *Agrp* and *Pomc* mRNA levels were measured at sacrifice and no significant effect of TTP2515 on gene expression was noted (results not shown).

**Table:** **Table 1.** Effects of TTP2515 on hormone levels and liver weight in rats receiving icv AgRP infusion.

	Saline+Water	AgRP+Water	AgRP+TTP2515	Saline+TTP2515
Leptin (ng/ml)	2.7±0.4	21.9±0.8*	18.5±4.2*	2.3±0.3
Insulin (ng/ml)	2.7±0.6	12.6±2.0*	9.7±2.2*	1.6±0.3
Plasma Glucose (mg/dl)	162.1±4.5	170±7.0	159.3±8.0	150.6±4.5
Liver Weight (g)	14.1±0.7	25.0±1.1*	21.8±1.7* †	13.7±0.5
Total T3 (ng/dl)	49.4±3.2	40.5±4.0	40.1±4.6	44.7±4.5

All measurements reported at sacrifice in *ad lib* fed rats. Values are mean ± SE.

* p<0.05 all groups;

† p<0.05 vs AgRP+Water.

### Experiment 3: Effects of TTP2515 on Metabolic Parameters in DIO Mice

#### Experiment 3a

**Figure 3:**
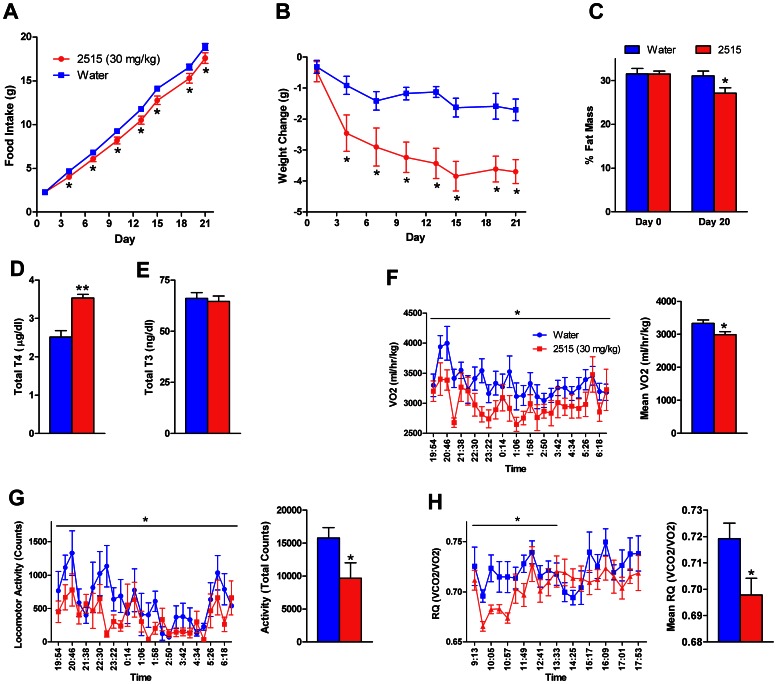
Effects of TTP2515 on metabolic and calorimetry parameters in DIO mice. (**A–E**) DIO mice (16 weeks on 45% fat diet) received TTP2515 (30 mg/kg) or water twice daily via oral gavage. (**A,B**) TTP2515 treatment significantly decreased food intake and body weight gain. (**C**) Percent fat mass was significantly lower in TTP2515 treated mice at the end of the study. (**D**) TTP2515 treated mice had elevated total T4 levels. (**E**) Total T3 levels were similar between groups. (**F–H**) In a separate experiment DIO mice (45% fat diet for 15 weeks) received TTP2515 (30 mg/kg) or water twice daily via oral gavage. (**F,G**) Mean oxygen consumption and total activity were significantly decreased during the dark cycle of day 2 in TTP2515 treated mice. (**H**) Mean respiratory quotient was significantly decreased during the first half of the light cycle of day 3 in TTP2515 treated mice. **p<0.0001, *p<0.05 vs. water.

DIO mice were gavaged with TTP2515 (30 mg/kg) or water twice daily for 21 days. Cumulative food intake and body weight gain were significantly decreased compared to water-treated mice (**Fig. 3A,B**). On day 20, the TTP2515-treated mice had significantly reduced percent fat mass compared to water treated mice (p<0.05, **Fig. 3C**), lost a greater amount of fat during treatment vs. the water group (−2.6±0.4 g vs −0.5±0.2 g, p<0.001), and tended to have lower absolute fat grams after treatment (12.0±0.9 g vs 9.8±0.8 g, p = 0.08). No difference in lean mass was observed between groups after treatment (**Table 2**). Mice received their last dose of TTP2515 or water at 800 h on day 21 and were sacrificed 6 h later. Insulin levels tended to be lower in TTP2515 treated mice after a 6 h fast on day 15 of the study (p = 0.14) and both insulin (p = 0.13) and leptin levels (p = 0.13) at sacrifice tended to be lower in TTP2515 treated mice (**Table 2**). At sacrifice, total T4, but not T3, levels were significantly elevated by TTP2515 treatment (p<.0001, **Fig. 3D,E**). No significant difference in *Pomc* or *Agrp* mRNA in the mediobasal hypothalamus was detected.

**Table 2:** Effects of TTP2515 in DIO and leptin-deficient mice.

	Water	TTP2515
**Exp 3a: DIO mice**		
Lean Mass (Day 20) (g)	19.8±0.4	19.9±0.6
Glucose-Day 15 (ng/dl)	161.0±4.4	164.0±3.9
Insulin-Day 15 (ng/ml)	2.4±0.5	1.6±0.2
Insulin (ng/ml)	2.0±0.3	2.1±0.7
Leptin (ng/ml)	25.0±2.6	23.3±3.8
Liver Weight (g)	1.3±0.1	1.3±0.1
Liver Weight as a % of Body Weight (%)	3.5±0.1	3.8±0.1
**Exp 4: Leptin-deficient mice**		
Glucose (ng/dl)	452.9±34.6	479.7±34.5
Insulin (ng/ml)	6.1±1.3	6.6±0.7
Liver weight (g)	2.9±0.1	2.3±0.2*
Liver Weight as a % of Body Weight (%)	5.7±0.2	5.3±0.3
Body Temperature (°C)	32.8±0.3	32.6±0.2

All measurements reported at sacrifice in *ad lib* fed mice unless otherwise indicated.

Values are mean ± SE.

* p<0.05 vs water.

#### Experiment 3b

A separate group of DIO mice were placed in the calorimetry system and received either TTP2515 (30 mg/kg) or water for 3.5 days. Cumulative food intake (10.5±0.5 g vs 10.0±0.6 g) and body weight change (−0.1±0.2 g vs −0.5±0.4 g, Water vs TTP2515) were not significantly different in DIO mice receiving TTP2515 treatment for 3.5 days. During the first two nights of treatment, VO2, EE and physical activity were reduced in TTP2515-treated mice compared to the water-treated mice. On the first night, this was only a tendency (**Table 3**), however this effect was significant during the night of day 2 (**Table 3**, **Fig. 3F, G**). Yet, these effects were transient, as VO2, EE and activity were not different during the nights of days 3 and 4 (**Table 3**). Furthermore, as the observed decrease in VO2 and EE was likely driven by decreased activity, VO2 and EE were divided by activity to produce a “relative” value. Relative VO2 and EE were significantly higher in TTP2515 treated mice during the dark cycle on day 1 (VO2∶374.2±54.4 vs 231.2±22.6 ml/hr/kg per counts; EE: 2.1±0.3 vs 1.3±0.1 W kg^−1^ per counts, p<0.05) and day 2 (VO2∶428.1±8.3 vs 227.0±22.2 ml/hr/kg per counts; EE: 2.4±0.5 vs. 1.3±0.1 W kg^−1^ per counts, p<0.05) but not different between groups on days 3 and 4. Respiratory quotient (RQ) was not different during days 1–2, however during the light cycle of day 3, mean RQ tended to be lower in TTP2515 treated mice compared to the water group (p<0.08), and was significantly lower during the first half of the light cycle (p<0.05, **Fig. 3H**).

**Table:** **Table 3.** VO2, EE and activity during the dark cycle in calorimetry experiments.

	Average VO2 *(ml/kg/hr)*		Average EE *(W kg^−1^)*		Locomotor activity *(total counts)*	
Exp 3b: DIO mice	*Water*	*TTP2515*	*Water*	*TTP2515*	*Water*	*TTP2515*
Day 1: Dark Cycle	3277±112	3096±80	18.3±0.6	17.3±0.5	15462±1952	10094±1858
Day 2: Dark Cycle	3335±103	2992±92*	18.6±0.6	16.7±0.5*	15746±1571	9646±2366*
Day 3: Dark Cycle	3170±119	3058±81	17.7±0.7	17.1±0.5	13773±1541	12710±2671
Day 4: Dark Cycle	3208±89	3232±119	17.9±0.5	18.0±0.7	13561±1924	12181±2593
**Exp 4: Leptin-deficient mice**						
Day 6 Dark Cycle	2291±110	2007±68*	13.2±0.6	11.5±0.4*	4271±620	2809±539
Day 12 Dark Cycle	2072±51	1924±74	11.8±0.3	11.0±0.4	4517±964	3029±413
Day 13 Dark Cycle (Fasting)	1406±36	1262±57	7.7±0.2	6.9±0.3	5328±916	1852±298**
**Exp 5: Fed/fasted lean BL6 mice**						
Day 1 Dark Cycle	2963±63	2824±46	17.1±0.4	16.3±0.3	10625±1772	6249±811*
Day 2 Dark Cycle	2979±72	2894±28	17.1±0.4	16.7±0.2	8592±1264	7223±983
Day 3 Dark Cycle (Fasting)	2440±106	2420±52	13.5±0.6	13.3±0.3	19086±4939	10664±2218
Day 4 Dark Cycle (Fasting)	2589±108	2450±44	14.3±0.6	13.5±0.3	22196±5323	9410±1773*
**Exp 6b: AgRP KO mice**						
Day 3 Dark Cycle	3232±127	2936±50*	18.7±0.8	16.9±0.3*	18331±4147	8137±1410*
Day 6 Dark Cycle	3111±134	2961±83	18.0±0.8	17.1±0.5	20756±6871	7769±1131
Day 9 Dark Cycle	3069±119	2913±63	17.7±0.7	16.8±0.4	21404±7107	8209±1377

In all VO2 and EE measurements above, adjustment for activity rendered VO2 and EE either significantly higher in the TTP2515 group or equivalent between groups. W, Watts; Values are mean ± SE.

** p<0.01,

* p<0.05 vs water.

### Experiment 4: Effects of TTP2515 on Metabolic Parameters in Leptin-deficient Mice

**Figure 4:**
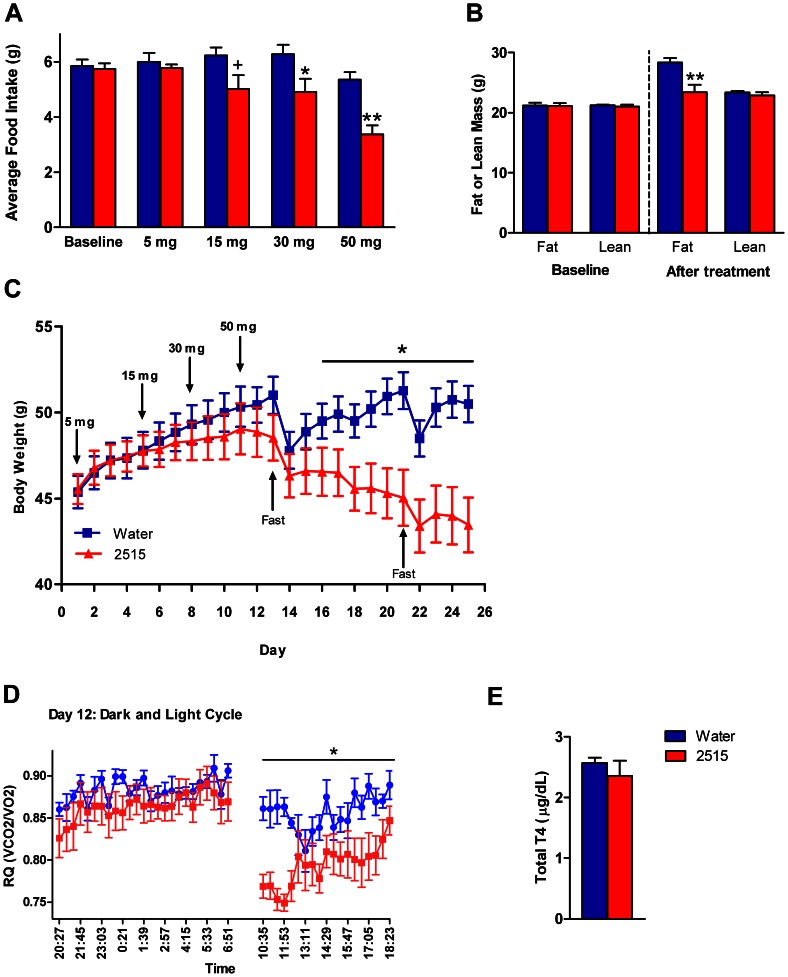
Effects of TTP2515 on metabolic and calorimetry parameters in leptin-deficient mice. Leptin-deficient mice received either TTP2515 at increasing doses (5–50 mg/kg) or water twice daily via oral gavage. (**A**) Food intake tended to be lower in TTP2515 treated mice during the 15 mg/kg treatment period and was significantly lower during the 30 and 50 mg/kg treatment periods. (**B**) Fat mass was significantly decreased after 24 days of TTP2515 treatment, while lean mass was not different between groups. (**C**) Body weight was significantly lower in TTP2515-treated mice on the 50 mg/kg dose. (**D**) Immediately upon starting the 50 mg/kg dose, mean respiratory quotient was significantly decreased in TTP2515 treated mice during the light cycle. (**E**) Total T4 levels at sacrifice were unchanged between groups. +p = 0.06, *p<0.05, **p<0.01 vs water.

Leptin-deficient *ob/ob* mice maintained on a normal chow diet received either TTP2515 at increasing doses (5–50 mg/kg) or water treatment for 25 days. During days 1–13, mice were placed in the calorimetry system. In *ob/ob* mice, average daily food intake was decreased with 15 mg (p = 0.06), 30 mg/kg (p<0.05) and 50 mg/kg (p<0.01) TTP2515 treatment (**Fig. 4A**); significant reductions in body weight were observed at the 50 mg/kg dose (p<0.05, **Fig. 4C**). No effects on VO2, EE or activity were observed during any of the dosing periods except transiently at the 15 mg/kg dose, where mean VO2 and mean EE (p<0.05) were decreased during the dark cycle of day 6, with a trend for decreased activity (p = 0.10, **Table 3**). However, this decrease in VO2 and EE was not evident if adjusted for activity. Mean RQ was significantly lower in 50 mg/kg TTP2515 treated mice, however this was not noted at lower doses. The decrease in RQ was observed during the light cycle immediately upon starting the 50 mg/kg treatment on day 11 and was again noted on day 12 (**Fig. 4D** **)**.

Mice were fasted during the evening of day 13. Fasting did not significantly increase activity in water treated mice, however TTP2515 treatment during fasting decreased activity (p<0.01), VO2 and EE (p = 0.05) compared to water treatment (**Table 3**). If adjusted for activity, VO2 and EE were increased in the TTP2515 group (p<0.05). TTP2515 treated mice also lost significantly less weight during the fast vs. water treated mice (Water −2.3±0.1 vs. TTP2515 −1.5±0.2 g, p<0.01), which may be secondary to the decreased activity observed. Mice were refed the next morning in homecages outside the calorimetry system. Both groups refed to a similar degree at 22 h (Water 4.0±0.5 vs TTP2515 3.0±0.5 g, p = 0.17) and gained a similar percentage of their original body weight during refeeding (Water 42.8±3.4% vs TTP2515 25.6±15.9%, p = 0.31).

On day 21, mice were fasted overnight and a GTT performed the next morning revealed no difference in glucose tolerance between groups (data not shown). MRI performed on day 24 showed that TTP2515 treated mice had significantly lower fat mass compared to water treated mice (p<0.001, **Fig. 4B**); lean mass was not different between groups. Mice received their last dose of TTP2515 or water on day 24 at 2200 h and were sacrificed at 1100 h on day 25. At sacrifice, liver weight (p<0.05) was significantly reduced in TTP2515 treated mice (**Table 2**), however plasma glucose, insulin, body temperature (**Table 2**) and total T4 levels (**Fig. 4E**) were not different between groups.

### Experiment 5: Effects of TTP2515 on Metabolic Parameters in Fed and Fasted Lean BL6 Mice

**Figure 5:**
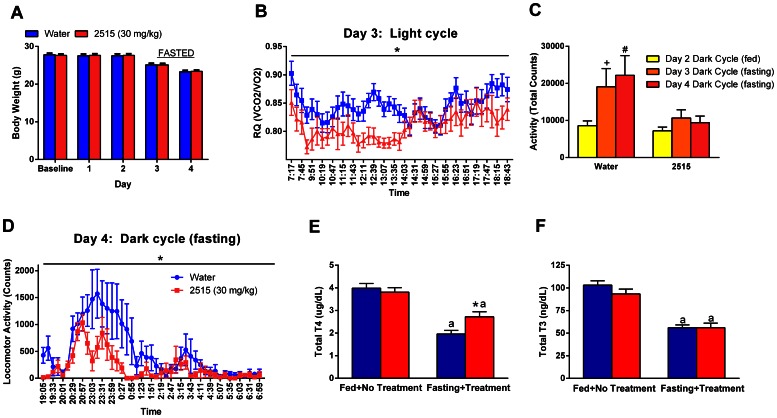
Effects of TTP2515 on metabolic and calorimetry parameters in lean fed and fasted mice. Lean, chow-fed mice received either TTP2515 (30 mg/kg) or water twice daily via oral gavage during both *ad lib* feeding and fasting. (**A**) Body weight was not different between groups during either the fed or fasted periods. (**B**) During the light cycle of day 3, mean RQ was significantly suppressed in TTP2515 treated mice. (**C**) During fasting, total activity was elevated in water treated, but not TTP2515-treated mice. (**D**) During the second night of fasting, total activity was significantly lower in TTP2515 treated mice compared to the water group. (**E**) After fasting, total T4 levels were significantly suppressed in both groups, however total T4 levels were significantly higher in TTP2515 treated mice. (**F)** Total T3 levels were suppressed by fasting and fasted levels were similar between TTP2515 and water treated mice. ^+^p = 0.06, ^#^p<0.05 vs day 2 Dark Cycle (fed); *p<0.05 vs water, a p<0.05 vs respective fed treatment.

Lean mice on a breeder chow diet were treated with either TTP2515 (30 mg/kg) or water for a total of 4 days in the calorimetry system. Mice were fed *ad lib* and then fasted. In fed mice, TTP2515 treatment did not decrease daily food intake (Water 3.0±0.0 vs. TTP2515 3.1±0.2 g) or attenuate weight gain, and weight loss was similar between groups during fasting (**Fig. 5A**). No significant differences in VO2 or EE were noted during days 1 and 2. However, a small but significant decrease in activity in TTP2515 treated mice was noted during the dark cycle on day 1 (p<0.05); this effect was transient as it was no longer observed during the dark cycle of day 2 (**Table 3**). Although no differences in RQ were observed during the dark cycle, on day 3 mean RQ was significantly lower during the light cycle in TTP2515 mice (p<0.05, **Fig. 5B**). As mice were fasted from the evening of day 3, it is unknown if this reduction in RQ would persist. During fasting, a significant increase in activity was noted in the control water mice during the dark cycle that was not observed in TTP2515 mice. Activity in the water group during fasting was elevated versus the fed state (day 2) as well as compared to TTP2515 mice during fasting on day 4 (p<0.05, **Fig. 5C, D**). No difference in VO2 or EE was observed between the TTP2515 and water groups during fasting (**Table 3**). Total T4 and T3 levels were similar before treatment in these two groups of mice that were subsequently treated with TTP2515 or water and fasted. After fasting, total T4 and T3 levels were significantly suppressed in both groups. However, after fasting, T4 levels were significantly higher in TTP2515 compared to water treated mice, while total T3 levels were similar between groups (p<0.05**,** **Fig. 5E, F**).

To further investigate if TTP2515 modulates metabolic parameters in lean mice, we conducted a separate experiment in which 50 mg/kg TTP2515 or water was administered twice daily via oral gavage to lean, breeder-chow fed WT mice (n = 7/group) for 7 days. During this time daily and cumulative food intake (WT 21.8±0.6 vs. TTP2515 21.1±0.4 g, p = 0.59) and daily and cumulative body weight gain (Water −0.4±0.2 vs. TTP2515 −0.2±0.3 g, p = 0.62) were similar between groups and both groups had similar fat and lean mass after 7 days of treatment (data not shown). The last dose of TTP2515 or water was administered at 2000 h on day 6 and a blood sample obtained at 0900 h on day 7 revealed a significant increase in total T4 levels in the TTP2515 group compared to the water group (5.3±0.2 vs. 4.3±0.2 µg/dl, p<0.01).

These experiments demonstrate that TTP2515 does not reduce food intake or body weight gain in lean, chow-fed mice at doses up to 50 mg/kg. However, chronic TTP2515 treatment did increase total T4 levels in both fasted and fed lean mice. We also observed that TTP2515 can transiently suppress physical activity and prevent fasting-induced increases in physical activity.

### Experiment 6: Effects of TTP2515 on Metabolic Parameters in AgRP KO and WT Mice on a 45% Fat Diet and Calorimetry Parameters in AgRP KO Mice

#### Experiment 6a

**Figure 6:**
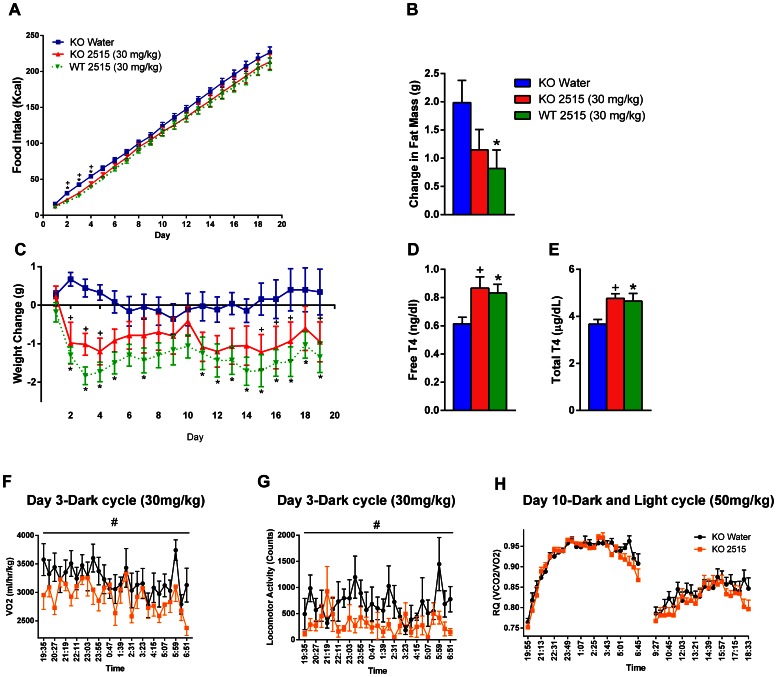
Effects of TTP2515 on metabolic and calorimetry parameters in AgRP KO and WT mice on a 45% high fat diet. (**A–F**) AgRP KO and WT mice received TTP2515 (30 mg/kg) and AgRP KO mice received water twice daily via oral gavage; all groups were on a 45% fat diet. (**A**) During days 1–4, there was an overall suppressive effect of TTP2515 treatment on food intake (p<0.05). Food intake was lower on days 2–4 in the WT TTP2515 and KO TTP2515 groups compared to the KO water group (p<0.05) (**B**) WT TTP2515 mice gained significantly less fat mass during treatment vs. the KO water group, however, this was not true for the KO TTP2515 group (p = 0.12). (**C**) Overall, there was a significant suppressive effect of treatment on cumulative body weight change (p = 0.01); body weight gain was more frequently significantly lower in the WT TTP2515 vs. the KO water group, rather than in the KO TTP2515 vs. the KO water group (**D,E**) Total and free T4 levels were elevated in both TTP2515 treatment groups compared to the KO water group. (**F–H**) AgRP KO mice were administered either TTP2515 (30 mg/kg days 1–7; 50 mg/kg days 7–10) or water twice daily. (**F,G**) On day 3 during the dark cycle, oxygen consumption and activity were significantly lower in TTP2515–treated mice. (**H**) RQ was not different between groups during the light or dark cycle at the 50 mg/kg dose (showing day 10, representative graph of this timeperiod). +p<0.05 KO Water vs KO TTP2515; *p<0.05 KO Water vs WT TTP2515; ^#^p<0.05 vs water.

AgRP KO mice were treated with either TTP2515 (30 mg/kg) or water and WT mice received TTP2515 (30 mg/kg) for a total of 19 days while receiving a 45% HFD. At the start of the experiment, AgRP KO TTP2515 (28.8±0.5 g) and AgRP KO water (29.3±0.6 g) groups weighed significantly less than the WT TTP2515 group (32.3±1.2 g); however, baseline food intake was similar between groups. Overall during the entire experiment, there was no significant difference in food intake between groups. Since the peak effect was observed during the first four days of treatment, cumulative food intake was analyzed during this period and was found to be significantly lower in the WT TTP2515 and the KO TTP2515 group, compared to the KO water group during days 2–4 (p<0.05, **Fig. 6A**). Overall, there was a significant suppressive effect of treatment on cumulative weight change between the groups (p = 0.01**,** **Figure 6C**). Post-hoc analysis revealed that body weight gain was more frequently significantly lower in the WT TTP2515 group vs. the KO water group, rather than in the KO TTP2515 group vs. the KO water group. By day 19 of the experiment, the cumulative weight loss in the WT TTP2515 group was −1.35±0.40 g vs. a gain of 0.34±0.59 g in the KO water group, while the KO TTP2515 group lost −0.95±0.52 g. After treatment, the WT TTP2515 group gained significantly less fat compared to the KO water group (p<0.05), while the KO TTP2515 group did not (p = 0.12, **Fig. 6B**). Lean mass was similar between all groups after treatment. At sacrifice, both total and free T4 levels were higher in WT and KO TTP2515 treated mice compared to KO water treated mice (p<0.05, **Fig. 6D, E**).

#### Experiment 6b

Lean AgRP KO mice maintained on a breeder chow diet were administered either TTP2515 (30 mg/kg *days 1–7*, 50 mg/kg *days 7–10*) or water in the calorimetry system. During the 30 mg/kg treatment period, no significant effects on average daily food intake (Water 3.2±0.2 vs TTP2515 2.9±0.1 g) or body weight were observed (Water 27.2±0.8 vs TTP2515 26.6±0.5 g). During the 30 mg/kg dosing period, RQ was transiently lower during the dark cycle on days 1 and 2 (p<0.05, data not shown) in the TTP2515 group and was not different during any other point in the experiment (**Fig. 6H**). On day 3, VO2, EE and activity were transiently lower in TTP2515 treated mice during the dark cycle (p<0.05, **Fig. 6F, G** **; Table 3**); this is similar to what has been observed in lean, DIO and *ob/ob* mice in *Exp 3–5*. Subsequently a tendency for reduced activity persisted without any change in VO2 or EE (**Table 3**). Adjustment for activity showed that relative VO2 and EE were equivalent between groups during the dark cycles. No other differences in calorimetry parameters were observed during the 30 mg/kg treatment period. During the 50 mg/kg treatment period, average daily food intake was similar between groups (Water 3.0±0.1 vs. TTP2515 2.8±0.1 g, p = 0.30) and body weight was not different (Water 27.3±0.9 vs. 26.5±0.5, p = 0.40), although the TTP2515 treated group tended to gain less weight by day 10 (Water 0.2±0.2 vs. TTP2515 −0.4±0.2 g, p = 0.05). At the 50 mg/kg dose, there were no significant differences in any calorimetry parameters but activity tended to be lower in TTP2515 treated mice during the dark cycle on days 8–10, without any change in VO2 or EE (**Table 3**). If adjusted for activity, relative VO2 and EE were either not significantly different or tended to be higher in the TTP2515 group vs. water on these days (Day 8 p<0.05, Day 9 p = 0.05, Day 10 p = 0.32).

### Experiment 7: Effects of TTP2515 on Hyperphagia and Metabolic Parameters After an Acute Switch to a Very High Fat Diet in C57BL6/J Mice and AgRP KO Mice

#### Experiment 7a

**Figure 7:**
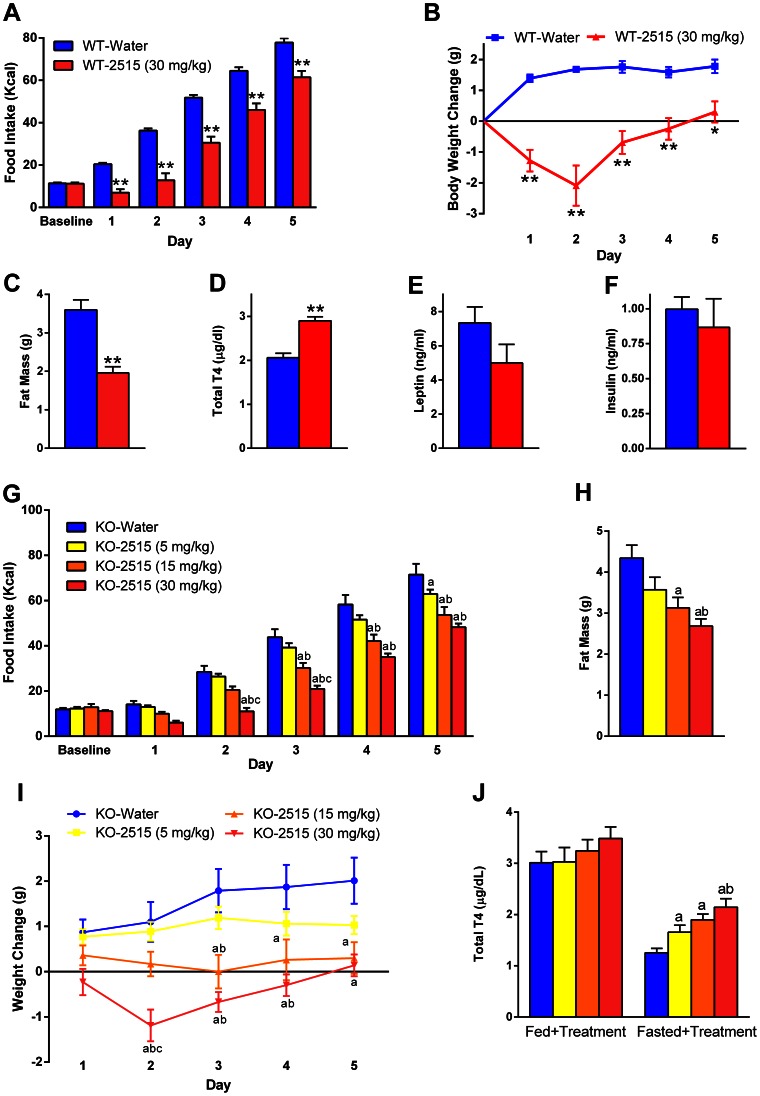
Effects of TTP2515 in WT or AgRP KO mice acutely switched to a very high-fat diet. (**A–F**) Mice were switched from breeder chow to a 60% fat diet and started treatment with TTP2515 (30 mg/kg) or water twice daily. (**A–C**) Cumulative food intake, cumulative weight gain and fat mass were all significantly lower in TTP2515 treated mice. (**D**) Total T4 levels were significantly higher in TTP2515 treated mice. (**E**) Leptin levels tended to be lower in TTP2515 treated mice (p = 0.12). (**F**) Insulin levels were similar between groups. (**G–J**) In a separate experiment, AgRP KO mice on a 10% fat chow diet were switched to a 60% fat diet and started treatment with TTP2515 (5, 15, or 30 mg/kg) or water twice daily. (**G,I**) Cumulative caloric intake and cumulative weight gain were dose-dependently lower in TTP2515 treated mice compared to the water group. (**H**) Fat mass was lower in TTP2515 treated mice at both the 15 and 30 mg/kg doses vs. water. The 5 mg/kg group tended (p = 0.05) to have lower fat mass than the water group. (**J**) After 5 days of treatment, total T4 levels were equivalent between groups, however fasting and continued treatment revealed an increase in total T4 levels. *p<0.05, **p<0.01 vs water; *a*p<0.05 vs water, *b*p<0.05 vs 5 mg/kg, *c*p<0.05 vs 15 mg/kg.

Lean, BL6 mice maintained on breeder chow were all switched to a 60% fat diet and simultaneously started treatment with either TTP2515 (30 mg/kg) or water. After switching to a HFD, water treated mice increased their caloric intake (p<0.0001, baseline vs. day 1) while the TTP2515 treated mice actually decreased their caloric intake (p<0.05, baseline vs. day 1). Furthermore, daily food was significantly lower in TTP2515 compared to water treated mice during days 1–2 (p<0.0001), however by day 3 daily food intake was similar between groups. Cumulative caloric intake was significantly lower in TTP2515 vs. water treated mice during the entire treatment period (p<0.0001, **Fig. 7A**). Water treated mice gained weight after switching to the higher fat diet; oppositely, TTP2515 treated mice lost weight (p<0.01, **Fig. 7B**). A body composition measurement on day 4 revealed that TTP2515 treated mice had significantly lower fat mass compared to water treated mice (p<0.0001, **Fig. 7C**), while lean mass was equivalent between groups (Water 19.6±0.5 vs. TTP2515 19.7±0.4 g). At sacrifice, leptin (p = 0.12) and insulin levels were not significantly different between groups, however total T4 levels were higher (p<0.0001) in TTP2515 treated mice (**Fig. 7D–F**). No significant difference in *Agrp* mRNA in the mediobasal hypothalamus was detected.

#### Experiment 7b

AgRP KO mice were all switched from a 10% fat chow diet to a 60% very HFD and simultaneously started treatment with either water or TTP2515 at 5, 15, or 30 mg/kg doses. On day 1, after switching AgRP KO mice to the HFD, the water group’s daily caloric intake increased slightly, although not significantly. In contrast, the 30 mg/kg TTP2515 treatment group consumed significantly fewer calories vs. baseline (p<0.001 vs. baseline values). On day 1, the 15 mg/kg and 30 mg/kg TTP2515 groups dose-dependently consumed fewer calories than the water group (p<0.05 water vs. both 15 and 30 mg/kg groups, p<0.05 15 mg/kg vs 30 mg/kg dose). Daily caloric intake was also significantly lower on days 2 and 3 in the 15 and 30 mg/kg groups (p<0.01) vs. saline. Cumulative food intake is depicted in **Fig. 7G**; post-hoc analyses revealed that cumulative food intake was significantly lower in the 30 mg/kg and 15 mg/kg TTP2515 groups compared to the water group from day 2 and 3 onwards, respectively (p<0.05). Cumulatively, water treated mice gained weight after switching to the higher fat diet; oppositely, TTP2515 treated mice lost weight (p<0.01, **Fig. 7I**). MRI on day 5 revealed that fat mass was significantly lower in the 15 and 30 mg/kg groups compared to the water treated group (p<.01, **Fig. 7H**). Lean mass was similar between groups (data not shown). After 5 days of treatment, total T4 levels were not different between groups; however fasting with treatment revealed a significant and dose-dependent increase in total T4 levels in TTP2515 treated mice (**Fig. 7J**).

These experiments indicate the TTP2515 treatment yielded a potent, anorexic effect in both AgRP KO and WT mice when switched to a HFD. The anorexic effect persisted for approximately 2–3 days, after which daily food intake was equivalent between the TTP2515 and water groups. However, cumulative food intake and weight gain continued to be lower in TTP2515 compared to water treated mice.

### Experiment 8: Effects of TTP2515 on Refeeding and T4 After Fasting in AgRP KO and WT Mice

#### Experiment 8a

**Figure 8:**
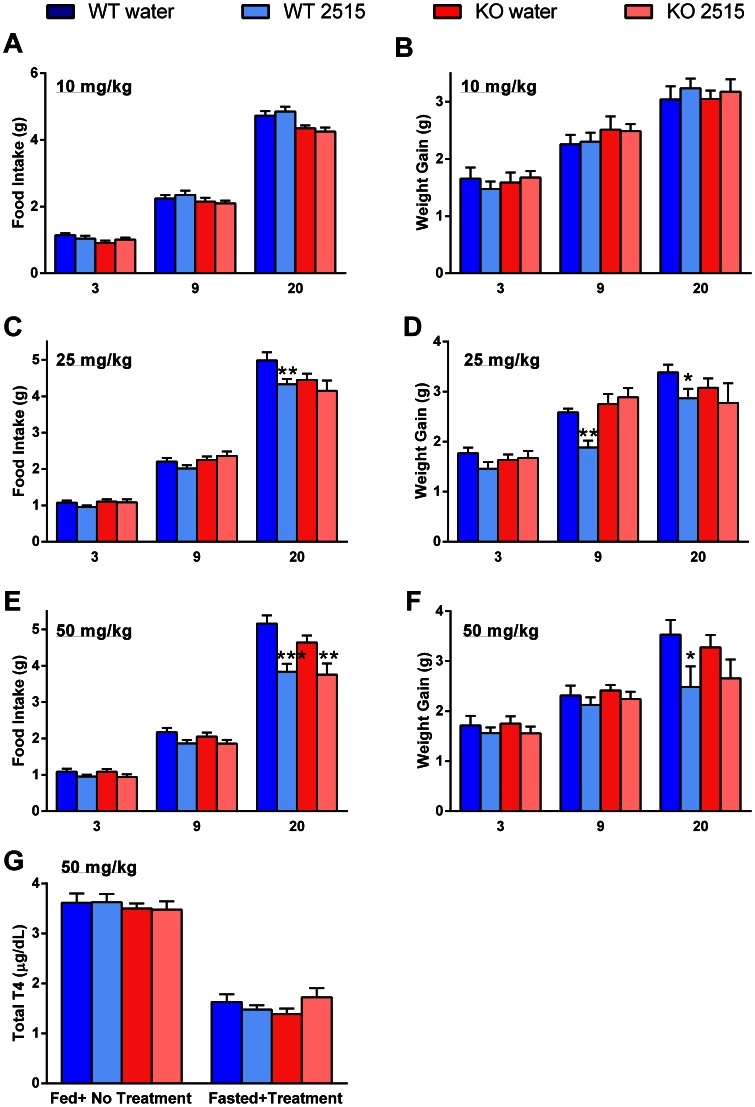
Effects of TTP2515 on refeeding and T4 after fasting in AgRP KO and WT mice. (**A–F**) AgRP KO and WT mice were fasted for 24 h and received either water or TTP2515 at 10, 25, or 50 mg/kg in three different experiments. (**A,B**) At the 10 mg/kg dose, no differences in food intake or body weight gain were observed. (**C,D**) At the 25 mg/kg dose, there was an overall significant attenuation in food intake (p<0.05) and weight gain (p<0.01) in the WT TTP2515 vs. the WT water group. Post-hoc analysis revealed that this was significant at the 20 h for food intake and at 9 and 20 h for weight gain. No differences in food intake or weight gain were observed between the KO TTP2515 vs KO water group. (**E**) At the 50 mg/kg dose, there was an overall significant attenuation in food intake in both the WT TTP2515 (p<0.01) and KO TTP2515 (p<0.05) groups vs. their water controls. Final cumulative food intake at the 20 h timepoint was significantly less in both TTP2515 groups vs. their water groups. (**F**) At the 50 mg/kg dose, final cumulative weight gain at 20 h was significantly less only in the WT TTP2515 vs WT water group. (**G**) A separate group of mice was fasted and blood samples were obtained before and after treatment. No difference in total T4 levels were observed before or after treatment. ***p<0.0001, **p<0.01, *p<0.05 vs respective water control.

In three separate experiments, mice were fasted for 24 h and received a single dose of TTP2515 (10, 25, or 50 mg/kg) or water the next morning and were refed 1 h later. Food intake and body weight measurements were obtained at 3, 9 and 20 h after refeeding and a second dose of TTP2515 or water was administered at the 9 h timepoint. In all experiments, baseline food intake was similar between groups, however WT mice were approximately 3 g heavier than AgRP KO mice (p<0.001). The 10 mg/kg dose did not attenuate refeeding or body weight gain in either KO or WT TTP2515 treated mice compared to their respective water counterparts at any of the timepoints measured (**Fig. 8A, B**). At the 25 mg/kg dose, there was an overall significant reduction in food intake (p<0.05) and body weight gain (p<0.01) in the WT TTP2515 group compared to the WT water group, however this was not observed between the KO TTP2515 and KO water group. At the 9 h timepoint WT TTP2515 treated mice gained less weight than WT water treated mice (p<0.01, **Fig. 8C, D**), but no difference between KO water and KO TTP2515 treated mice was observed. At the 20 h timepoint, WT TTP2515 mice refed less (p<0.01) and gained less weight (p<0.05) than WT water treated mice, however this effect was not observed between the KO water and KO TTP2515 groups. At the 50 mg/kg dose, there was a significant overall effect of TTP2515 treatment on feeding in both the WT (p<0.01) and KO (p<0.05) groups compared to their water controls (**Fig. 8E**). Post-hoc analyses showed that at the 20 h timepoint, both WT (p<0.0001) and KO (p<0.01) TTP2515-treated mice refed significantly less than their water controls. With respect to weight gain, only final cumulative weight gain at 20 h was significantly less in the WT TTP2515 vs WT water group; this difference was not significant between the two KO groups (**Fig. 8F** **)**.

Thus the 25 mg/kg TTP2515 dose attenuated refeeding and weight gain specifically in WT, but not AgRP KO mice. At the 50 mg/kg dose, TTP2515 treatment attenuated refeeding in both AgRP KO and WT mice, suggesting that this higher dose may elicit non-specific effects.

#### Experiment 8b

AgRP KO and WT mice were fasted and received either TTP2515 at 50 mg/kg or water during the fast. Three doses of TTP2515 were administered with the last dose at 2000 h; blood samples were collected at 0700 h the next day. Baseline total T4 levels before fasting and treatment were not significantly different between groups. After fasting and TTP2515 treatment, total T4 levels were not significantly different between groups (**Fig. 8G**).

## Discussion

This study shows that TTP2515 functions *in vitro* as an AgRP inhibitor, and when administered orally in rats, was able to attenuate the effects of centrally infused AgRP. When studied in cells overexpressing the human MC4-R, TTP2515 blocked AgRP-mediated antagonism of α-MSH-induced increases in cAMP levels. TTP2515 did not affect the MC4-R either alone or in the presence of α-MSH, consistent with an interaction with AgRP and not directly with the MC4-R. Prior studies with a related compound failed to block SHU9119’s antagonistic effects, demonstrating selectivity for AgRP. When administered to rats by oral gavage, TTP2515 blocked AgRP-induced increases in food intake, weight gain and adiposity, as well as suppression of total T4 levels. The effects on food intake and body weight gain were observed in two different experiments using either single icv AgRP injections or continuous icv AgRP infusion. These effects were not observed in icv saline-treated rats in either experiment and animals receiving TTP2515 consumed food and appeared to behave normally.

Subsequent experiments utilized TTP2515 in mouse models to investigate its effects on endogenous AgRP. Although TTP2515 did not reduce food intake, body weight gain or adiposity in lean wild-type mice at either the 30 mg/kg or 50 mg/kg dose, in DIO mice, TTP2515 effectively reduced these parameters. This is consistent with the expected actions of an AgRP inhibitor resulting in increased MC4-R activity. These effects are similar to what has been reported in DIO mice overexpressing MSH [6]. However, the effects of TTP2515 on food intake and body weight could not be solely attributed to AgRP inhibition as an attenuation in these parameters was also observed in DIO AgRP KO mice treated with TTP2515. There was a tendency for WT TTP2515 treated mice to lose more weight than KO TTP2515 treated mice, but this was not significant. However, WT TTP2515 mice gained significantly less fat mass during the experiment compared to KO water mice, while the KO TTP2515 and water treated mice gained more similar amounts, suggesting that TTP2515 is somewhat more effective in WT mice in preventing fat mass accretion, compared to KO DIO mice. Furthermore, a significant decrease in RQ during the light cycle was also noted in TTP2515 treated DIO mice, consistent with predicted changes in adiposity and energy partitioning that might be expected with AgRP inhibition. The effects of TTP2515 on RQ noted in WT mice during the light cycle were not observed when KO mice were treated with either 30 mg/kg or 50 mg/kg TTP2515. This data suggests that the effects of TTP2515 on energy partitioning may be specific for AgRP antagonism and is consistent with findings from a report on AgRP KO mice [18]. In contrast to what would be predicted, TTP2515 treated DIO mice displayed a transient decrease in VO2 and EE which could be explained by the concomitant unexpected decrease in physical activity observed. In fact, if adjusted for activity, VO2 and EE were no longer suppressed in TTP2515 treated mice. These transient effects on VO2, EE and activity were also evident in TTP2515 treated AgRP KO mice and thus could not be attributed to AgRP antagonism. These transient effects on VO2, EE and activity were not observed in *ob/ob* mice, however this was likely due to their inherently low baseline levels. Thus, TTP2515 treatment reduced food intake, body weight gain and adiposity in both AgRP KO and WT mice on a HFD, but not in mice on a chow diet and reductions in adiposity and RQ observed in HFD fed mice appeared to be more pronounced in WT than KO mice.

TTP2515 was extremely effective in preventing weight gain in obese leptin-deficient mice on a chow diet; leptin-deficient mice are reported to have elevated hypothalamic AgRP expression [12]. Significant, dose-dependent reductions in food intake and body weight gain as well as decreases in adiposity and RQ were noted in these mice. It is unknown if these effects are specific for AgRP antagonism as we did not study leptin-deficient AgRP KO mice. However, the same 50 mg/kg dose that potently reduced food intake, weight gain, and fat mass accretion in *ob/ob* mice failed to modulate any of these parameters in lean wild-type mice on a chow diet. These data suggest that the effects observed in *ob/ob* mice could be due to AgRP antagonism.

TTP2515 was quite effective under fasting conditions where AgRP expression in the hypothalamus is increased [11], [13], [31]. Dose response studies show that TTP2515 can attenuate rebound feeding in mice at both a 25 mg/kg and 50 mg/kg dose. At 25 mg/kg TTP2515 attenuated rebound feeding and weight gain in WT mice, but not in KO mice, and at the 50 mg/kg dose, TTP2515 attenuated cumulative body weight gain in WT mice, but not KO mice, indicating that these effects appear to be relatively specific for AgRP antagonism. As attenuated rebound feeding at the 50 mg/kg dose was observed in both KO and WT mice, this suggests that higher doses may yield non-specific effects. Furthermore in lean BL6 mice, TTP2515 treatment attenuated fasting-induced increases in activity. This is in agreement with previous data that has shown that activating the AgRP neuron drives food-seeking behavior [19]. Unfortunately, activity was not studied in AgRP KO mice during fasting.

Several lines of evidence demonstrate a role for the melanocortin system in modulating the hyperphagic response initially observed after switching mice to a high diet. First, switching BL6 mice from a normal chow diet to a higher fat diet (42–45%) produces an initial hyperphagic response that is quickly downregulated [30], [34]; however MC4-R knockout mice are unable to downregulate their food intake after initiation of a higher fat diet [30], [34]. Secondly, AgRP increases fat intake [35], [36], while MTII decreases preferential fat consumption [36]–[38]. Furthermore, Agouti-overexpressing (A^y^) mice and MC4-R KO rats have an increased preference for fat [39], [40]. In addition, *Agrp* mRNA levels decline after mice are switched to a HFD [41]. We therefore hypothesized that TTP2515 would be particularly effective in attenuating the acute effects after initiation of a HFD. Introduction of the 60% HFD to mice that simultaneously started TTP2515 treatment significantly reduced caloric intake; this reduction was significant compared to both baseline chow diet intake and versus water treatment. Mice treated with TTP2515 recover after approximately 3 days and resume normal daily food intake, however lower cumulative food intake and body weight gain persisted in TTP2515 treated mice after 5 days. This effect is quite remarkable given that mice maintained on a breeder chow diet and administered TTP2515 treatment are unaffected by the compound. This data suggests that TTP2515 has a potent effect on fat intake. However, surprisingly, TTP2515 was equally effective when the same experiment was conducted in AgRP KO mice. Thus, this effect is not due to AgRP antagonism. The dose-responsive effect observed in KO mice suggests that TTP2515 may bind to a similar, perhaps yet unknown, target which strongly promotes fat intake. As the effect on fat intake was initiated rapidly and with such potency, it suggests that TTP2515 could potentially have a local effect in the gut in the presence of increased dietary fat, which could lead to the suppression of food intake. Regardless of the mechanism, these results are of interest as compounds that promote decreased dietary fat intake may have clinical utility.

It was also predicted that an AgRP inhibitor would increase thyroid hormone levels under certain conditions given the role that AgRP plays in regulating the thyroid axis and mediating the fasting induced suppression of T4 and T3 levels [8], [16], [17]. Additionally, in one study, AgRP KO mice were reported to have higher total T4 and T3 levels [18]. In the current study icv AgRP suppressed plasma T4 levels (as expected), however this suppression was prevented by TTP2515 treatment. TTP2515 also increased total and free T4 levels in lean and DIO mice when animals were treated for at least 4 days. T4 was also increased after TTP2515 treatment in fasted mice, however this was only observed in an experiment when the drug was administered for 4 days and not when administered for a shorter duration (1.5 days). Of note, TTP2515 had no effect on T3 levels at any time in any experiment. T4 measurements are notoriously subject to technical problems with a variety of assays, especially in the presence of drugs that can affect binding proteins and assay performance, hence the validity of these T4 measurements may come into question. In any case, the effects of TTP2515 on T4 levels are not specific for AgRP antagonism; we showed that both TTP2515 treated KO and WT mice had elevated free and total T4 levels compared to KO mice receiving water treatment.

Previous studies have utilized genetic models of AgRP deletion or AgRP neuronal ablation to uncover AgRP’s effects on energy balance and metabolism. However, the interpretation of these studies is complicated by developmental compensation and the loss of other neurotransmitters from AgRP neurons [18], [21], [23]. This is the first report of a pharmacological inhibitor of the AgRP peptide that could be used to help elucidate the role of AgRP in regulating energy homeostasis. The effects of TTP2515 are most prominent under conditions in which AgRP is known to be elevated such as during fasting and with leptin deficiency [11]–[13], [31]. TTP2515 also affected feeding and metabolic responses after acute or chronic exposure to a HFD, conditions that can be modulated by the melanocortin system. Although TTP2515 demonstrated some clear off-target effects in AgRP KO mice, there was some relative specificity to the effects on adiposity and RQ as well as on refeeding after fasting, which are consistent with AgRP’s orexigenic and metabolic function. The cause of the off-target effects are at present unclear. It is of interest that most of the non-AgRP mediated effects observed are still consistent with what would be expected with stimulation of the melanocortin system. It may be that TTP2515 also binds to a peptide of similar function to AgRP that modulates food intake, energy balance and preference for fat intake. Although Agouti is a potential candidate as it possesses 25% sequence homology to AgRP [12], in mice it is expressed in the skin, functions in a paracrine manner and is not known to regulate energy balance unless ectopically expressed as in mice with the A^y^ mutation [42]. However, interaction with other related peptides or non-peptides is clearly possible. Future studies should be directed at uncovering the mechanism by which TTP2515 yields such potent effects favoring negative energy balance, even in the absence of AgRP.

The rationale for using an AgRP inhibitor, rather than an MC4-R agonist, to increase melanocortin signaling relates to the fact that AgRP neuronal projections are more restricted to brain areas regulating energy balance; hence this inhibitor will affect only a subset of MC-Rs, compared to the wide distribution of brain MC4-Rs that would be affected by an MC4-R agonist [25], [26]. Although MSH agonists are effective, they have been associated with a number of side effects including increased blood pressure, due to the extensive distribution of brain MC-Rs [29]. Furthermore, an AgRP inhibitor has the potential to be utilized in a therapeutic capacity, particularly under conditions in which AgRP levels are elevated. In humans, this might be utilized optimally during diet and weight loss or during weight maintenance after weight loss has been achieved. Additionally, the potent effect of TTP2515 on fat consumption suggests that this peptide could also be advantageous before consuming a fat-rich meal, to attenuate HFD food intake.

In summary TTP2515 functions *in vitro* as an AgRP inhibitor, and when administered orally exerts numerous metabolic effects in rats and mice. Many of these effects are consistent with AgRP inhibition, yet in some cases are evident in AgRP null mice indicating off target effects of this drug. TTP2515 is most effective in obese models including DIO and *ob/ob* mice, as well as during consumption of a HFD and does not cause weight loss in lean mice on a low fat diet. Thus the usefulness of this drug in treating human obesity deserves further exploration. Future studies are needed to define the AgRP dependent and independent mechanisms by which this drug exerts its effects on energy balance.

## Supporting Information

Table S1 **Streamlined Description of Experiments.**
(DOC)
