# Supplementary material for: Effects of AgRP Inhibition on Energy Balance and Metabolism in Rodent Models
Source: PLoS One. 2013 Jun 6;8(6):e65317. doi: 10.1371/journal.pone.0065317 (PMC3675096; doi:10.1371/journal.pone.0065317)
Supplement: Table S1 — Streamlined Description of Experiments. (DOC) [file pone.0065317.s001.doc]

Supplementary Table 1: Streamlined Description of Experiments

| **Experiment Number and Description** | |
| --- | --- |
| 1. ***In vitro* studies in cells overexpressing the MC4-R**, testing the effects of the compound TTP2515 on MSH-induced production of cAMP. | 1. **Studies in rats examining the ability of TTP2515 to antagonize the effects of exogenous AgRP. A:** Initial experiment testing the effects of oral TTP2515 on AgRP-induced increases in food intake and body weight after two single ICV AgRP injections. **B:** In-depth investigation of the effects of oral TTP2515 on AgRP-induced increases in food intake, body weight, adiposity, and relevant metabolic hormones during a one-week continuous ICV infusion of AgRP. |
| 1. **Effects of TTP2515 in an established mouse model of diet-induced obesity. A:** Effects of TTP2515 on body weight, food intake, adiposity and levels of metabolic hormones in **DIO mice.** **B:** Effects of TTP2515 on energy expenditure, substrate utilization and activity in **DIO mice.** | 1. **Dose-dependent** **effects of TTP2515** on body weight, food intake, adiposity, energy expenditure, substrate utilization, activity and levels of metabolic hormones in **leptin-deficient *ob/ob* mice,** with known elevations in hypothalamic AgRP levels. |
| 1. Effects of TTP2515 on body weight, food intake, energy expenditure, substrate utilization, activity, and levels of metabolic hormones in **fasted lean mice** (with high levels of hypothalamic AgRP) **vs fed lean mice.** | 1. **Comparison of effects of TTP2515 in AgRP KO and WT mice** to determine the specificity of TTP2515 for AgRP. **A:** Effects of TTP2515 on body weight, food intake, adiposity and levels of metabolic hormones in **DIO AgRP KO and WT mice**. **B:** **Dose-dependent** effects of TTP2515 on energy expenditure, substrate utilization and activity in **lean AgRP KO and WT mice.** |
| 1. Effects of TTP2515 on food intake, weight gain, adiposity and metabolic hormone levels after simultaneously **switching to a very high fat diet** and starting TTP2515 treatment. **A:** Lean WT mice. **B:** Lean AgRP KO mice. | 1. **Effects of TTP2515 during fasting. A:** Effects of TTP2515 on food intake and weight gain after **fasting at 10, 25, and 50 mg/kg doses in both AgRP KO and WT mice. B:** Effects of acute TTP2515 treatment on thyroid hormone levels after fasting. |
